# Information Circulation in times of Ebola: Twitter and the Sexual Transmission of Ebola by Survivors

**DOI:** 10.1371/currents.outbreaks.4e35a9446b89c1b46f8308099840d48f

**Published:** 2018-08-28

**Authors:** Celine Morin, Ida Bost, Arnaud Mercier, Jean-Pierre Dozon, Laetitia Atlani-Duault

**Affiliations:** Media Studies Department, HAR/IRMECCEN, Paris Nanterre University, Paris, France; Anthropology Department, LESC, Paris Nanterre University, Paris, France; Media Studies Department, Institut Français de Presse, Paris Assas University, Paris, France; CEMAF (IRD - EHESS), Fondation Maison des Sciences de l'Homme, Paris, France; Fondation Maison des Sciences de l'Homme, Université Sorbonne Paris Cité, Paris, France; IRD, CEPED, Université Sorbonne Paris Cité, Paris, France; School of Public Health, Columbia University, New York, United States

**Keywords:** ebola, information circulation, sexual transmission, survivors, Twitter health crisis

## Abstract

Introduction: The 2013-2015 outbreak of Ebola was by far the largest to date, affecting Guinea, Liberia, Sierra Leone, and secondarily, Nigeria, Senegal and the United States. Such an event raises questions about the circulation of health information across social networks. This article presents an analysis of tweets concerning a specific theme: the sexual transmission of the virus by survivors, at a time when there was a great uncertainty about the duration and even the possibility of such transmission.

Methods: This article combines quantitative and qualitative analysis. From a sample of 50,000 tweets containing the words “Ebola” in French and English, posted between March 15 and November 8, 2014, we created a graphic representation of the number of tweets over time, and identified two peaks: the first between July 27 and August 16, 2014 (633 tweets) and the second between September 28 and November 8, 2014 (2,577 tweets). This sample was divided into two parts, and every accessible publication was analyzed and coded according to the authors’ objectives, feelings expressed and/or publication type.

Results: While the results confirm the significant role played by mainstream media in disseminating information, media did not create the debate around the sexual transmission of Ebola and Twitter does not fully reflect mainstream media contents. Social media rather work like a “filter”: in the case of Ebola, Twitter preceded and amplified the debate with focusing more than the mainstream media on the sexual transmission, as expressed in jokes, questions and criticism.

Discussion: Online debates can of course feed on journalistic or official information, but they also show great autonomy, tinged with emotions or criticisms. Although numerous studies have shown how this can lead to rumors and disinformation, our research suggests that this relative autonomy makes it possible for Twitter users to bring into the public sphere some types of information that have not been widely addressed. Our results encourage further research to understand how this “filter” works during health crises, with the potential to help public health authorities to adjust official communications accordingly. Without a doubt, the health authorities would be well advised to put in place a special watch on the comments circulating on social media (in addition to that used by the health monitoring agencies).

## Introduction

Social media today are key sources of data that can be used for understanding health crises. They are, for users, a space and a hub for circulating information 1^,^^2^^,^^3^ and, for researchers, a tool for understanding how health crises are experienced and perceived 4^,^^5^^,^^6^^,^^7^^,^^8^^,^^9^. The fact that users may share information or promote rumors and misinformation 10^,^^11^^,^^12^^,^^13^^,^^14^ offers unprecedented opportunities for studies to address the links between the public’s posting of information (original thoughts, sharing and questioning of material from both mainstream media and official sources) and levels of fear and panic 15^,^^16^^,^^17^^,^^18^^,^^19^^,^^20^**.** As a free application with contents restricted to 140 characters, the networking and microblogging platform Twitter is one of the most popular social media. During the most recent Ebola outbreak, approximately 100,000 tweets containing the word “Ebola” were posted each day before September 30, 2014 – the date that the first Ebola diagnosis on American soil was reported. Once news of this diagnosis had “broken”, this number multiplied by eleven, first reaching around 870,000 tweets by the end of the day, and 1.6 million tweets the day after, on October 1st 2014, across Europe, North America, Africa, Asia and Australia 21. Similar spikes occurred following official announcements of Ebola cases in the United States 8, despite the actual small number of cases.

Most information posted on social media during a health crisis has been shown to emanate from the accounts of news organizations and health authorities, but also from humorous accounts and even celebrity ones 11**.** Recent studies have called on health authorities to monitor Twitter, to identify the most active and influential accounts, and to understand or even to adopt social media platforms and rhetoric 11^,^^20^^,^^22^**.** This is all the more important that it is now evident that Twitter has become as an important public space for criticism of health authorities: Roberts et al. 20 showed that official health information had some prominence in mainstream media reports but less in social media. In other words, public health officials have failed to impose their narrative during the crises.

However, if it is clear health authorities need to learn from social media users, we are yet to understand precisely how these users contributed to shape the debates that emerged online. By analyzing in detail the sentiments, questions and critiques that users expressed in tweets, on the one hand, and their reading and circulation of mainstream media articles and official announcements on the other, this study provides new insights on the social conversation that arose during the most recent Ebola crises. A more precise comprehension of the diverse and sometimes contradictory perceptions of these crises is crucial to a better understanding of the circulation of information during a humanitarian crisis.

Our study combines qualitative and quantitative analysis and focuses on a specific topic discussed on Twitter: the sexual transmission of Ebola by survivors. This topic was controversial at the time of the outbreak, as the nature of such transmission was not widely understood. An overview of studies on the subject shows few mentions 21^,^^23^^,^^24^^,^^25^ until the first transmission was confirmed on March 20, 2015, after which numerous articles were published 26^,^^27^^,^^28^^,^^29^^,^^30^^,^^31^^,^^32^. It is now known that Ebola virus genetic material can linger more than 500 days in semen 33. Subtil et al. 34 estimate that the probability of Ebola RNA is up to 31.6% at 3 months and falls to 0.7% at 18 months. Same decreasing trend was noticed by Sow et al. 35. Moreover, the variability of Ebola RNA presence in semen has been highlighted by Keita et al. with patients testing positive, then negative, and then positive again 36. Although the World Health Organization (WHO) published a Twitter post on March 23rd 2014 stating that sexual transmission of Ebola was possible via “infected semen” for “up to seven weeks after clinical recovery”, this news, although an official pronouncement, had little impact on Twitter. Only one user repeated its content the same day. Our data shows that it was only at the end of July that the issue began to gain importance online. We examined two significant peaks identified in a sample of 50,000 tweets that were posted between March 15 and November 8, 2014. The peaks had significantly different contexts. The first one started on July 27 and ended on August 16, 2014 (633 tweets). It relates to the fast spread of the virus to countries like Nigeria, and its consequences such as the ban that South Africa and Kenya (and indeed Liberia) imposed on travelers from West Africa, as well as the preparations of the United States and Europe to deal with a potential outbreak. The second peak started on September 28 and ended on November 8, 2014 (2,577 tweets). It followed the dying of the first Ebola patient diagnosed on American soil, Thomas Eric Duncan. At the same time as these peaks were occurring, the total number of cases was declining and the WHO was hoping to announce that Nigeria and Senegal were free of Ebola after 42 days with no new cases.

Our results show that while mainstream media continued to strongly influence the information in circulation, online debates became autonomous on the matter of the sexual transmission by survivors. This led to the expression of criticisms of official institutions and about the definition of Ebola as an STD, with strong references to AIDS. At a first level of analysis, the results confirm the significant role played by mainstream media in diffusing information. But at a second level, and contrary to what is usually understood in the literature, our data show that Twitter does not fully reflect other media content. Rather, it works more like a filter that highlights issues that have been published by mainstream media. By analyzing the circulation of information on Twitter and the responses of its users (be these responses criticisms, jokes or expressions of doubt), this article not only complements existing scientific literature but also provides insight into the social debate that occurred around the Ebola epidemic. It analyzes which information was in circulation and how it was summarized, reformulated, criticized or simply rejected online. In addition, this study examines how Twitter users can, during a debate on a subject as sensitive as the sexual transmission of the virus, act as complementary to official sources by autonomously selecting and highlighting an issue that was treated as a side-note by the media. More broadly, this article attempts to show how an analysis of posts sent through Twitter during outbreaks might be used in order to adjust official communications.

## Materials and Methods

**Data collection**

Web content mining methods were applied to the tweets with steps similar to those demonstrated by Yoon and Bakken 37: selection of keywords; importation of data; preparation of data; analysis of data; content analysis (topics and sentiments) and finally, data interpretation. Inspired by previous studies 37^,^^38^^,^^39^^,^^40^, we first used French and English keywords to identify relevant tweets. These two languages are widely used by people from the different countries affected by Ebola (Guinea, Liberia, Sierra Leone, and secondarily, Nigeria, Senegal and the United States). Semiocast, our partner company for social media analysis, provided the first sample of 50,000 tweets containing the words “Ebola”, posted between March 15 and November 8, 2014, allowing us to preprocess the data 40. For ethical and technical reasons, it was impossible for us to locate Twitter users and categorize them according to this parameter. All tweets were extracted without any personal data to comply with ethical rules for studies on public health issues. We used a topic model to identify the latent issue of sexual transmission by survivors 41: two researchers analyzed this sample and created a list of 17 keywords, keeping the best possible equivalence between French and English, in order to extract all tweets, with the exception of retweets without modification (Table 1).

**Figure:**
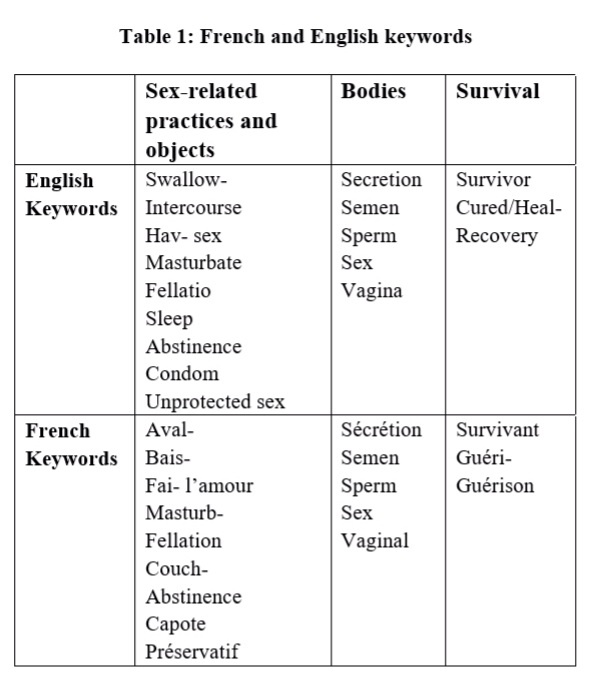
**Table 1:** French and English keywords.

These two researchers then manually sifted through this sample and removed irrelevant tweets. In cases of uncertainty as to classification, tweets were discussed collectively. When necessary, we used the Twitter search application to understand the discussion context. The final corpus numbered almost 6,000 tweets, making it impossible to carry out a full analysis. We created a graphic representation of the number of tweets over time, and identified two peaks: the first between July 27 and August 16, 2014 (633 tweets) and the second, between September 28 and November 8, 2014 (2,577 tweets). An information watch on all health institutions’ Twitter accounts (ONG, CDC, WHO, Public Health Agency of Canada) was also set up during the research to monitor their announcements and reactions from the Twittersphere.

**Classification**

This sample was divided into two parts, and each researcher coded half the data. When the classification of a tweet was unclear, the tweet was discussed collectively. The coding categories were not defined beforehand but elaborated according to the characteristics of the data, in order to take the polysemy into account.

The tweets were clustered according to the authors’ objectives (see, for example, reference 42). Five categories were created:

- Asking for information;
- Challenging and rectifying information;
- Providing information;
- Giving an opinion;
- Other.

We also indicated tweets expressing feelings, such as anger, laugher or fear. We took into account only tweets with hashtags (Such as #weregonnadie, #funfact, #fail, #whathasobamadonetous, etc.), emoticons or clear indications about a specific feeling (Such as “we all dead”, “It's only getting worse”, “LOL” “hahahahaa”, “LMAO”, “That's a lie!”, etc.).

As in previous studies (such as the study carried out by Hughes and Palen 43), we took any URL links into account to analyze the “networked interplay” (20) between information from mainstream media and tweets. Every accessible publication was analyzed and coded according to publication type. Five categories of publication were identified:

- Newspaper articles from mainstream media with an editorial byline (The New York Times, lemonde.fr, The Washington Post, etc.);
- Publications from public organizations (ONG, CDC, the WHO, Public Health Agency of Canada, etc.);
- Publications from blogs, social networks, discussion forums and video sharing websites (Youtube, Twitter, Facebook, MSN, etc.);
- Scientific articles;
- Other (specialized websites about health, medical care, travel, or online encyclopedia).

## Results

**The relative influence of mainstream media and official organizations on Twitter**

98.7% of the tweets collected are written in English. But French tweets profiles are similar in terms of organization and general content, and there appeared to be no significant difference between English and French tweets.

Relatively few tweets express emotion. From the full corpus, 7.5% of the tweets clearly express anxiety or fear, 4.4% express laughter and 1.4% express irritation and anger. Some users show interest in their followers and 4.2% of the tweets provide advice:

- Ebola can be transmitted through sperm too now!?? Ladies, DO NOT town! (August 13, 2014)
- Ebola can last in semen for up to 3 months. For you singles don't trust nobodyyyy (September 30, 2014)
- Ebola can live in semen for, 3 months wrap it up kids (November 1st, 2014)

A minority of the textual messages in tweets request information (4.1%), express an opinion (4.1%), or either challenge or correct information (0.6%). As is consistent with previous studies, the majority (more than 90%) only provide information.

A large proportion of the tweets (48.6%) contain at least one URL (1,572 out of 3,234 tweets). Among them, 466 tweets (29.6%) are drafted similarly and contain only one headline and one URL (and sometimes the publication’s name). This format is automatically provided by numerous publication websites:

- TIL that when males are cured of ebola virus, they can still transmit the virus in their semen for up to 2 mon... http://tinyurl.com/lzt6gks (August 4, 2014)
- Ebola Might Be Sexually Transmitted? http://wp.me/p2iq4c-9wL (October 3, 2014)
- Ebola outbreak: Survivors told to use condoms to prevent virus spreading http://ht.ly/2OKIWM (October 8, 2014)

It is very close to what has been described, in a study of the practices of Twitter users sharing information, as “slavish tweets.” This is a reference to the term “slavish copying” in copyright infringement cases, i.e. the Twitter users share information, staying totally neutral, without any comments 44.

Interestingly, the websites of health organizations are under-represented as sources of content. During the first peak, the WHO website accounts for only 9 links (5% of the URLs). During the second peak, in the ranking by website URL shared in the tweets, the WHO comes eighth (4% of all URLs) and CDC comes tenth (2.6% of all URLs). URLs link to a variety of media but the relative frequency with which these media are cited is very different: 1.4% (20 out of 1,417 valid URLs) are specialized sites and scientific articles, 5.8% (83 out of 1,419) are news aggregators, 8.1% (115 out of 1,419) are websites belonging to public organizations, 18.6 % (264 out of 1,419) are blogs, social networks or forums, and 65.9% (935 out of 1,419) are mainstream media.

The results reveal the strong influence of mainstream media. But mainstream media did not create the debate. Twitter interest in sexual transmission by survivors exceeded mainstream media coverage: the first publication dedicated to this topic, and shared on Twitter, was the article entitled “Ebola survivors can infect others with their sperm” published by the South African website Health 24 on August 13, 2014. Half of the 83 publications from blogs and mainstream media that specifically mention it, and were shared on Twitter, were published between October 5 and October 11, 2014. But the first peak on Twitter began at the end of July, showing that Twitter users did not wait until sexual transmission hit the headlines. They took this specific piece of information from a variety of sources, whether dedicated to a general presentation of the virus (Such as “Ebola virus disease” by Wikipedia), to the spread of the disease (Such as “Un modèle mathématique de la progression d'Ebola créé à l'EPFZ” published on October 8, 2014 by Romandie.com) or to survivors in general (Such as “Surviving Ebola: For those who live through it, what lies ahead?” published on July 30, 2014 by CBS). Some of these sources, such as “What Is Ebola? Six Things You Need to Know” (NBC July 29, 2014) or “How not to catch Ebola” (BBC October 7, 2014), contain only one or two sentences about sexual transmission. In other words, Twitter users deliberately selected this piece of information, mainly from mainstream media publications. The microblogging platform seems to work both as a filter of information but also as an alternative pool of information that users re-prioritize on Twitter.

**Figure:**
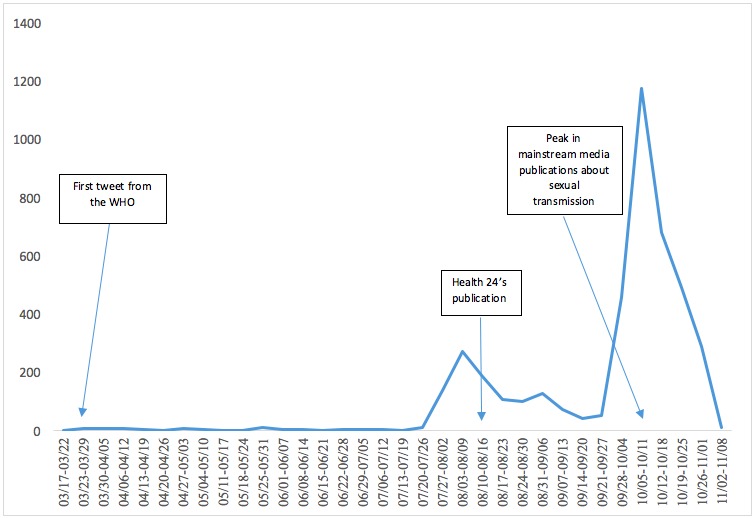
**Fig. 1:** Single tweets about sexual transmission by survivors, March–November 2014.

**Gaps between journalistic informations and conversations on Twitter**

Of the publications shared on Twitter via an URL, 17.3% are not dedicated to the sexual transmission, showing that tweet authors valued this piece of information more highly than the journalists. A question remains: what is the purpose of the publications shared on Twitter?

In general, the publications shared on Twitter via a URL express concern about the spread of the virus, and its arrival in Northern countries, as shown in the following tweet. A typical example states that “The threat of the disease traveling to a distant country is very real. A single infected passenger on an aircraft could spread the disease to another continent” (The Anti Media “Preventative Measures for Ebola in Case of an Outbreak” July 29, 2014). Some reflected a degree of anxiety. The authors emphasize the risk, the severity of the virus and the ability of Ebola to travel by air. This anxiety is not specific to forums, social networks or blogs, as the following example shows: “It's frightening, mysterious and yes, it could come here. […] No matter where you live, instant jet travel has made any infection capable of spreading worldwide, and that includes Ebola” says a journalist from NBC (“What Is Ebola? Six Things You Need to Know” July 29, 2014).

During the first peak (between July and August) numerous authors insist on the importance of avoiding bodily fluids, which are the main means of transmission. Avoiding sexual relationships with survivors appears to be the last recommendation. For example, in the article entitled “Ebola 101: The Facts Behind a Frightening Virus” (NPR, July 10, 2014), the sexual transmission of the virus is discussed in brackets: “**How does it spread?** Through close contact with infected blood, saliva, urine, stool and vomit. […] **Treatment**: […] Patients are declared Ebola-free if they don't show any symptoms for several days and if repeated tests for the virus in their bloodstreams come back negative. (The virus can still linger in semen for months […])

During the second peak (between September and November), the number of cases was decreasing and the post-outbreak situation was discussed. Sexual transmission of the virus seemed to be somewhat more prominent in the mass media:

- “Sex could keep the Ebola epidemic alive even after the World Health Organization (WHO) declares an area free of the disease” (Reuters “Male Ebola survivors told: Use a condom”, October 7, 2014).
- “Many of the survivors right now have detectable amounts of Ebola virus DNA in their semen or vaginal secretions—and many of them, presumably, are having sex” (The Daily Beast “Ebola Might Be Sexually Transmitted” September 4, 2014).

But the articles’ authors tend to mitigate their remarks regarding survivors. Many of them condemn the stigmatization of survivors:

- “The doctor has beaten the odds and survived Ebola, but he still has one more problem: the stigma carried by the deadly disease” (AP “Survivors of Ebola face second 'disease': stigma” April 27, 2014).
- “Ebola survivors are likely to be shunned and isolated by their communities” (NPR “Ebola 101: The Facts Behind A Frightening Virus” July 10, 2014)
- “These fears are almost entirely misplaced” (Mother Jones “How Long Does the Ebola Virus Survive in Semen?” October 8, 2014)

Only one article, entitled “Ebola survivor infects wife to death” (The Pluto Daily October 14, 2014), casts responsibility on a survivor, alleging that he killed his wife by imposing unprotected sexual intercourse upon her. Moreover, the author does not generalize this case, and he concludes by mentioning the important role of survivors, recalling that they “should be role models in society, as they should be the ones telling people about the realities of the disease rather than infecting others”.

Moreover, the articles’ authors present this information as a scientific uncertainty. 87.3% of the publications that focus on the sexual transmission of Ebola use the WHO as the main source of information, quoting a sentence from the WHO website, published on October 6. The WHO stressed the hypothetical nature of this information, stating that “more surveillance data and research are needed on the risks of sexual transmission, and particularly on the prevalence of viable and transmissible virus in semen over time.” Similarly, the media take into account this scientific uncertainty:

- “there is still the potential for them to spread the disease” (Health 24 “Ebola survivors can infect others with their sperm” August 13, 2014)
- “it's unclear just how great a risk the semen of surviving men poses in the weeks following their illness” (Mother Jones “How Long Does the Ebola Virus Survive in Semen? “ October 8, 2014)
- “No one is certain that the viral DNA is actually living, transmissible virus” (The Daily Beast “Ebola Might Be Sexually Transmitted” September 4, 2014).

In contrast, tweets do not always show such a cautious approach. Particularly, if we remove neutral tweets from the corpus, the remaining tweets reveal criticisms and questions about the authorities’ crisis management performance. Contrary to what one might expect, in general, tweets do not endorse the idea of survivors as a threat. The article entitled “Ebola survivor infects wife to death” (The Pluto Daily October 14, 2014) had limited uptake: it appears in only 4 links out of 1,236 valid URLs. In this sample, Twitter users show little interest in the survivors themselves: they express little blame and no compassion toward them. Only two tweets are direct accusations against survivors:

- @cnn African MEN are ebola death guns spewing the virus in their semen up to 6 months after infection. Small wonder it is spreading so fast (October 9, 2014)
- All Ebola bomb survivors are at jack in the box (October 9, 2014).

Tweets focus instead on the veracity of official information on the sexual transmission of the virus. Almost all the questions (131 tweets) ask for confirmation or additional information. Moreover, 40 tweets (85% of the tweets expressing anger) voice the idea that the risk posed by survivors’ ability to transmit the disease has not been communicated at all, or not on a wide enough scale, or contradicts previous official statements stressing that only sick people can transmit the disease:

- #ebola Why didn't CDC reveal that semen of Ebola patients transmits virus up to "7 weeks after clinical recovery"? http://www.phac-aspc.gc.ca/lab-bio/res/psds-ftss/ebola-eng.php (October 1st, 2014)
- @CDCgov Wrong! Men who survive #Ebola have it in their semen for 7 weeks, yet they aren't symptomatic then. #fail (August 2, 2014)
- Ebola can live 74 days in semen! The government is lying to you - you do not have to touch someone just be anywhere near where they've been (October 8, 2014).

A few salacious jokes reveal difficulty with understanding how that information has been obtained:

- Whoever is tasting the semen for Ebola is doing the work of angels. Wait, that's not how they test it? (October 2d, 2014)
- You tested

More pertinently, 10 tweets focus on condom use, which is denounced as ineffectual in the light of AIDS history:

- Survivors told to use condoms(why not refrain from sex for 3 mo instead,DONK!) http://www.enca.com/africa/ebola-survivors-told-use-condoms via @eNCAnews (October 8th, 2014)
- Ebola survivors told to use condoms? Great, it was such a success with HIV/AIDS wasn't it! http://www.independent.co.uk/news/world/africa/ebola-outbreak-survivors-told-to-use-condoms-to-prevent-virus-spreading-9782058.html (October 8, 2014)
- "ebola survivors should abstain from sex or use condoms for three months" seems idiotic to me, how'd you like to have that condom break (October 12, 2014)

Of the tweets expressing criticism, 54.3% attack the WHO, the CDC and the mainstream media. A further 8.6% blame President Obama and his government, while 37.1% of tweets are generic accusations and do not single out anyone in particular. Thus, as was found in research on the H1N1 epidemic 33, the CDC, the WHO and the mainstream media are the scapegoats of choice, as they are supposedly responsible for providing reliable information. Paradoxically, tweets requesting information are not generally addressed to them: governmental and health institutions (such as the WHO or the CDC) are mentioned in 30 tweets (22.4%) and only 18 tweets (13.4%) are sent to a mainstream media. The recipients of the tweets requesting information are mostly individuals (56%), half of whom have identified themselves as journalists, safety specialists or physicians. This behavior may be interpreted as a sign of mistrust in official institutions (names from personal account have been deleted)

**Understanding Ebola as an STD: the prevalence of AIDS**

Another question is much more developed in the tweets than in the publications: is Ebola an STD? Only a few articles question the type of the disease once it was suggested the virus might be transmitted by sexual relation. One journalist writes that “it is also an STD of sorts” (Quartz “Here are the 35 countries one flight away from Ebola-affected countries” July 30, 2014). Similar mentions included “Ebola is never going away, WHO warns, Ebola IS a sexually transmitted disease” according to a headline in Catholic Online (August 10, 2014) and “Yes, the Ebola virus is potentially a sexually transmitted disease” confirms The Daily Beast (“Ebola Might Be Sexually Transmitted” September 4, 2014).

Only four publications make a connection between AIDS and the sexual transmission of Ebola. The articles make it clear that the two diseases are widely feared and in both cases, condoms should be used to reduce the risk of transmission (Daily Beast “Ebola Might Be Sexually Transmitted” September 4, 2014; Government slaves “Ebola Might Be Sexually Transmitted?” no publication date; Pissin’on the roses “Until Gaëtan Dugas or Other Flying Rats Catch Ebola, The North American Risk Remains Low” April 1st, 2014). However, their authors do not significantly develop the comparison between the two diseases.

In contrast, the connection between Ebola and AIDS is more prevalent in the tweets. In 99 tweets (6% of the non-neutral tweets) Ebola is described as an STD or a parallel is drawn between AIDS and Ebola. These tweets rarely use quotations from media: the ideas expressed seem to come from the users themselves. From a qualitative point of view, the parallels between the two diseases are much more developed in the tweets than in the online publications. Twitter users often noted that the sexual mode of transmission is similar in both cases and condoms are necessary to prevent infection. But more specifically, AIDS is used to evaluate the gravity of Ebola. AIDS is thus treated as a kind of barometer, whose gravity is presumed known by all, used to gauge the dangers of this new virus:

- #Ebola, a sort of STD #virus it makes #AIDS look mild. Transmitted in semen as late as 61 days after patient recovery http://qz.com/242388/here-are-all-the-35-countries-one-flight-away-from-ebola-affected-countries/ (July 31st, 2014)
- People who recover from ebola still carry the virus, in sperm etc. #worsethanaids? (August 7, 2014)
- @Ask Ebola spread by semen? * This could be worse than AIDs. What color is the ribbon? Red? (October 13, 2014)

Although people make connections between AIDS and Ebola in general, they rarely discuss their own sexual activities and prefer to discuss general perspectives. Out of the whole corpus of tweets, only 151 tweets (5% of the single tweets) refer to daily life in the message or by using hashtags (#StayStrapped, #metalpantswithlockon, #Abstain, #WrapItUp, #SafeSex, etc.). Jokes reveal that sexual transmission is a difficult subject to speak about:

- "Ebola is not airborne mom!" -me "Ebola stays in sperm for two months!" - mom #onlymymom #ohgosh (October 18, 2014)
- This girl literally out of nowhere in class goes "did you know the Ebola virus can be spread through semen?" (August 6, 2014)
- Kid in my class: "Ebola is transmitted by semen so better keep your mouth closed." (October1st, 2014)

Moreover, joking about sexuality is used to transform a serious illness into a funny subject according to a cathartic logic, in order to reduce the anxiety associated with the Ebola name:

- Ebola can live in vaginal fluid for 3 months, Michael Douglas' head emerges from his wife's puss. *I beat cancer, I'll beat this.* #Ebola (October 8, 2014)
- "Male survivors may be able to transmit the disease via semen for nearly two months." that's a seriously long orgasm, folks. i want #Ebola (October 17, 2016)
- The 50 Best Sex Positions For An Ebola Outbreak: (50 pictures of people not having sex, because ebola can live in semen for 60 days) (August 4, 2016)

## Discussion

The study of Tweets confirms the influence of mainstream media, which represent a large majority of the publications shared on Twitter. In our corpus, Twitter was rarely used for disseminating an alternative media discourse. Interestingly, however, the microblogging platform does not reflect mainstream media contents in a servile manner. Users focused on the sexual transmission of Ebola months before the media coverage of this piece of information: the first peak began at the end of July 2014 on Twitter. Users attached greater importance to the sexual transmission of Ebola than the journalists did. In this sense, Twitter seemed to work more like a filter than like a booster.

Because of this particularity, some of the issues raised by the sexual transmission of Ebola are much more developed in the tweets – in jokes, questions and criticism of communication by the authorities - than in the publications shared. Even if a majority of tweets are neutral and only provide information, the feelings or questions expressed in them could help to identify misunderstandings during health crises.

## Limits

The analysis is limited somewhat by the fact that it is impossible (for reasons of scientific ethics) to identify the location and socio-economic profile of individual Twitter users. We only worked on the discourses revealed in the Tweets, and could not investigate social groups. Nor can we give a reliable explanation of why English tweets are more numerous than French tweets. We were unable to discover why interest in the sexual transmission of the virus by survivors began at the end of July, and why not before, with the first tweet of the WHO, or after, with the media coverage.

It is also worth mentioning that we started an analysis of the images contained in tweets, only to realize that very few of them actually referred to the sexual transmission or even to the disease. Ebola is rather used as a metaphor for everyday life. We also used emoticons and emojis to help classify tweets according to the emotions they convey, but further research on these discursive marks would help to further understand the rhetoric of users online.

Finally, information is not only shared on Twitter. Another study could highlight the differences between the use of Twitter and that of comments or internet publications (forums, online articles, other social networks such as Facebook, etc.).

## Conclusion

This article increases our knowledge of the relationship between Twitter and traditional media in times of epidemics. In contrast to previous studies that underlined how users rely on Twitter for sharing information from mainstream media (such as 1^,^^2^^,^^3^), our study shows that tweets combine information dissemination with emotional stances and critical views, leading to a new or greatly increased debate about a subject that was treated as insignificant by other media. In that sense, Twitter acts mostly as a filter of information as well as a space where it is reconstructed. In the “Twittersphere,” the issue of sexual transmission of Ebola by survivors was mostly voiced by citizens and not by mainstream media. They did so by emphasizing the potential danger of the transmission and by defining Ebola as a STD.

While many Twitter users tweet and retweet in a neutral way, merely sharing URL links from media or official sources, there is a strong active minority that otherwise uses its public speaking power. By expressing their fears and doubts, and using the argumentative resources of irony or comparison (over time, or with events considered similar), Internet users raise questions and express objections that the health authorities did not consider necessary to discuss. Without a doubt, the health authorities would be well advised to put in place very quickly, on the occasion of every health crisis, a special watch on the comments circulating on social media (in addition to that used by the health monitoring agencies). It could prove a uniquely useful way to identify, even in the form of weak signals, criticisms or worries to which it is better to give answers before the rumors increase.

Online debates can of course feed on journalistic or official information, but they also show great autonomy, tinged with emotions or criticisms. Although numerous studies have shown how this can lead to rumors and disinformation, our research suggests that this relative autonomy makes it possible for Twitter users to bring into the public sphere some types of information that have not been widely addressed. Our results encourage further research to understand how this filter works during health crises, and suggests the potential of such research (particularly by shedding light on what interests or alarms citizens) to help public health authorities to adjust official communications accordingly.

## Corresponding Author

Celine Morin: morin.celine@gmail.com

## Data Availability

Data are freely available: https://doi.org/10.6084/m9.figshare.6026399.v1; https://doi.org/10.6084/m9.figshare.6026405.v1.

## Competing Interests

The authors have declared that no competing interests exist.
